# Family Dynamics and Mental Vulnerability of Older Adults during the COVID-19: Evidence from A National Study in China

**DOI:** 10.1093/geroni/igaf122.2307

**Published:** 2025-12-31

**Authors:** Zhe Chen, Senhu Wang

**Affiliations:** National University of Singapore, Singapore, Singapore; National University of Singapore, Singapore, Singapore

## Abstract

Mental vulnerability poses a serious threat to older adults, especially during the COVID-19 pandemic, underscoring the need to understand protective factors that mitigate such risks. Intergenerational family relationships have been shown to play a pivotal role in safeguarding older adults’ well-being, yet limited research has specifically examined their impact on mental vulnerability during the pandemic. This study investigates how family dynamics influenced the mental vulnerability of older adults in China during the COVID-19 outbreak, utilizing data from the 2020 wave of the China Health and Retirement Longitudinal Study (CHARLS). Employing ordered logit regression models, we analyze the effects of adult children’s gender, education level, and the quality of intergenerational relationships on older parents’ mental vulnerability. Results indicate that older adults with daughters, children holding college degrees, or strong intergenerational relationships experienced significantly lower levels of mental vulnerability compared to those with sons, children with lower educational attainment, or weaker intergenerational ties. The subgroup analyses further reveal that these effects vary by the gender of the older parent and are more pronounced among those living with a partner, suggesting that adult children complement the protective role of spousal support. These findings underscore the importance of family in mitigating mental vulnerability among older adults and call for targeted social policies that strengthen the protective functions of families for older adults.

